# 2D-DIGE Proteomic Analysis of Changes in Estrogen/Progesterone-Induced Rat Breast Hyperplasia upon Treatment with the Mongolian Remedy RuXian-I

**DOI:** 10.3390/molecules16043048

**Published:** 2011-04-08

**Authors:** Zhong-Chao Wang, Du E, De-Ligen Batu, Ya-Latu Saixi, Bin Zhang, Li-Qun Ren

**Affiliations:** 1 Department of Experimental Pharmacology and Toxicology, School of Pharmaceutical Sciences, Jilin University, Changchun 130021, China; 2 Affiliated Hospital of Inner Mongolia University for the Nationalities, Institute of Mongolia and Western Medicinal treatment, Tongliao 028000, China

**Keywords:** 2D-DIGE, estrogen, proteomics, breast hyperplasia, Mongolian medicine, rat

## Abstract

RuXian-I has traditionally been used as a remedy for breast hyperplasia in the Inner Mongolia Autonomous Region of China. As a first step toward the investigation of biomarkers associated with RuXian-I treatment, a proteome-wide analysis of rat breast tissue was conducted. First, rat breast hyperplasia was induced by injection of estradiol and progesterone. After treatment with RuXian-I, there is a marked decrease in the hyperplasia, as can be shown by decreases in the nipple diameter and the pathological changes in breast. Subsequently, we used an approach that integrates size-based 2D-DIGE, MALDI-TOF/TOF-MS, and bioinformatics to analyze data from the control group, the model group and the RuXian-I treatment group. Using this approach, seventeen affected proteins were identified. Among these, 15 (including annexin A1, annexin A2, superoxide dismutase [Mn], peroxiredoxin-1, translationally-controlled tumor protein and α B-crystallin) were significantly up-regulated in the model group and down-regulated upon treatment with RuXian-I, and two (Tpil protein and myosin-4) have the opposite change trend. The expression of annexin A1 was confirmed using immunohistochemistry. The expression of superoxide dismutase (SOD) activity was confirmed biochemically. These results indicated that RuXian-I treats rat breast hyperplasia through regulation of cell cycle, immune system, metabolic, signal transduction, *etc.* The differential expressions of these proteins (annexin A1, superoxide dismutase [Mn], alpha B-crystallins and translationally controlled tumor protein, among others) were associated with occurrence and metastasis of breast cancer. These findings might provide not only far-reaching valuable insights into the mechanism of RuXian-I action, but also leads for prognosis and diagnosis of breast hyperplasia and breast cancer.

## 1. Introduction

Much research has been devoted to malignant lesions of the breast because breast cancer is the most common malignancy in women in Western countries; however, benign lesions of the breast are far more frequent than malignant ones. Breast hyperplasia is one of these lesions and is also one of the risk factors for breast cancer. The World Health Organization advocates early prevention and detection in order to improve breast cancer outcome, and these measures remain the cornerstone of breast cancer control. However, patients with breast hyperplasia are reluctant to undergo tissue resection because it is painful, often recurs, and does nothing to treat the underlying, causative endocrine imbalance. Instead, multiple applied estrogen or estrogen receptor antagonist agents, including tamoxifen, have been used. Although these drugs can relieve symptoms in patients, they have many side effects and complications, and their use also can lead to endocrine disorders [1]. Mongolian medicine plays an important role in Chinese ethnic medicine, but very little scientific evidence exists that supports consumption of Mongolian medicine herbal preparations in traditional medicine, so RuXian-I is only in clinical use in the Affiliated Hospital of Inner Mongolia University for the Nationalities as a traditionally drug, and lacks widespread application. Clinical observation has shown that it has high efficacy and a low incidence of side effects. RuXian-I treatment of breast hyperplasia in 400 patients showed that the cure rate was 98%. Multiple biological activities have been reported for the main components of RuXian-I. For example, *Herba Leonuri Japonici* has anti-inflammatory and antioxidant activity, suppresses oxidative stress and ameliorates hypercholesterolemia activity. However, the therapeutic mechanism of RuXian-I is not clear. Proteomics provide an important theoretical basis for the study of the pathogenesis of diseases and their mechanism and the discovery of drug targets. Two dimensional difference gel electrophoresis (2D-DIGE) can greatly reduce the inter-gel variation and excessive time/labor costs associated with standard 2-DE and can allow a more accurate qualitative and quantitative analysis [2,3]. There have been several reports comparing the expression profiles of proteins in breast cancer tissues with those in nontumor breast tissues [4,5,6]. However, expression profiles of proteins in breast hyperplasia have not been reported. In order to gain a better insight into the mechanism of RuXian-I’s efficacy, diagnosis, therapy, outcomes, and prognosis in breast hyperplasia, and the relation between breast hyperplasia and breast cancer, we used intramuscular estrogen and progesterone injections to induce rat breast hyperplasia and 2D-DIGE to study the proteins associated with RuXian-I treatment. Differentially expressed spots were identified by MALDI-TOF/TOF-MS, and changes in the expression of the proteins of interest were further validated using immunohistochemical and biochemical detection methods.

## 2. Results and Discussion

### 2.1. Histopathological observation of breast tissue by H&E staining

**Figure 1:**
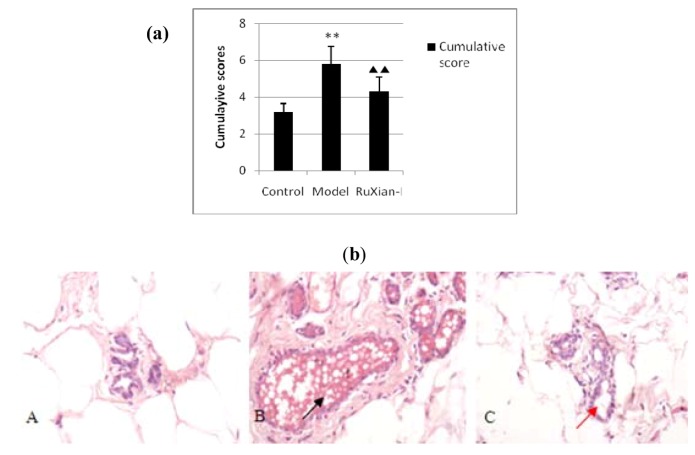
**(a)** Comparison of cumulative pathological grading scores in various groups. Score criteria, see 3.3. Values are means ± SEM. **P ≤ 0.01, compared with normal control group; ▲P ≤ 0.05, compared with model group. **(b)** H&E staining of mammary glands (magnification×400): (A) control group; (B) model group; (C) RuXian-I treatment group. The model group results were typical of breast hyperplasia, with an increased number of breast lobules, increased proliferation of the acini and ducts, and highly increased acinar secretion. The black arrows indicate acinar secretion of a high degree of expansion and strong. In the RuXian-I group, the numbers of lobular acini were less than the model group, the secretion into the acinis and the lumen volume was reduced. Some of the glands in these rats closely resembled normal rat mammary glands. See red arrows indicated.

The control group had the following phenotypes in various regions: in the duct, there was a lack of ectasia and of significant proliferation of epithelial cells, and an epithelial arrangement; the lobules were very small with 1–3 acini per lobule and no expansion into the acinar cavity; and there were no significant stromal hyperplasia. In the disease model group, the duct showed disorganized architecture: the ductal walls had layers of cells, most epithelial cells were irregularly arranged and there was a large amount of shedding epithelium and secretions in the lumen. This was accompanied by a significantly increased lobular volume and an increase in the number of lobular acinar per lobule (to 7–8/lobule) as well as significant expansion in the acinar chamber and stromal hyperplasia. However, no mammary tumors were observed. In the RuXian-I group, there was slight ectasia in the lobular ducts and mild epithelial hyperplasia, but the epithelial cell arrangement persisted. The ductal wall displayed no significant thickening, and there was a decrease in epithelium and secretions in the lumen. The lobular volume increased slightly, as did the number of acini (to 3–4/lobule); however, no significant stromal hyperplasia was observed in the RuXian-I group. In short, it can be shown that RuXian-I has high efficacy from Histopathological observation of breast tissue. The cumulative pathological grading scores of the breast tissues are shown in Figure 1(a), and the H&E staining is shown in Figure 1(b).

### 2.2. Comparison of nipple diameters in rats

**Figure 2:**
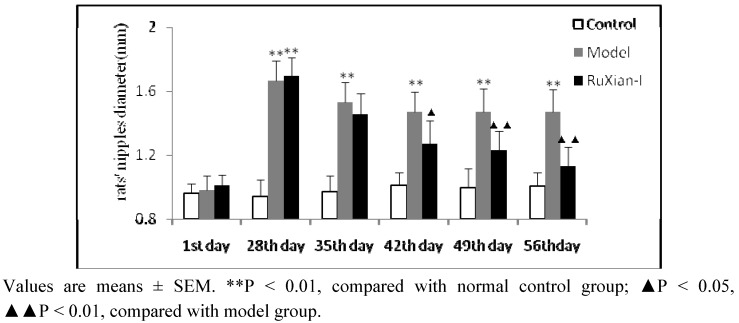
Comparison of nipples diameter in rats. The nipple diameters of the model group were larger than those of the normal controls, and the nipple diameters of the RuXian-I-treated were reduced significantly when compared to the model group.

Observation of the rat nipples and measurement of the diameter of the nipples may indirectly reflect breast hyperplasia, supporting the hypotheses that hyperplasia was successfully modeled here and that RuXian-I treatment was effective. Before hormone treatment, the rat nipples were white, small, soft, adherent to the skin, and close to invisible, and the nipple diameters were similar across groups. After establishment of the model, the nipples were become redness, swelling, larger and more visible than those of the normal control. Four weeks after administration, the nipple diameters of the model animals were still larger than those of the normal controls (P ≤ 0.01). After treatment with RuXian-I for 4 weeks, nipple redness and swelling was reduced significantly. The nipple diameters of RuXian-I groups were markedly smaller than those of the model group (P ≤ 0.01). The change in nipple diameters over time is shown in Figure 2.

Estrogens promote the proliferation of the breast duct and that a combination of estrogen and progesterone can promote the proliferation of breast has confirmed [7]. Estrogen and progesterone, in combination, can increase the risk for breast hyperplasia [8,9,10]. In this study, in order to simulate endogenously high levels of estrogen and estrogen/progesterone imbalance, rats were injected with estradiol (0.5 mg/kg/d) for 25 d followed by injection of progesterone (4 mg/kg/d) for 5 d. The results showed that the size of the nipples, breast lobules, and acinis increased significantly upon this treatment. The acinar and ductal epithelial hyperplasia, though variable, persisted for 30 days after the cessation of hormone injections, making this a good model for mammary hyperplasia in humans. Moreover, after treatment with RuXian-I, there is a marked decrease in this hyperplasia, as can be shown by decreases in the nipple diameter and the pathological changes in breast. 

### 2.3. Proteomic analysis to identify changes in protein expression stimulated by RuXian-I treatment in rat breast hyperplasia

#### 2.3.1. Analysis of Differentially Expressed Proteins

We use 2D DIGE to analyze the control group, the disease model group, and the RuXian-I treatment group for differential proteins expression in the mammary gland. After 2D DIGE, the Cy2, Cy3, and Cy5 channels of each gel were individually imaged, and the images were analyzed using DeCyder 5.0 software. Among 2,118 matched protein spots, we detected 43 differentially regulated protein features associated with hyperplasia, of which 27 were altered upon RuXian-I treatment. There were 22 spots that were significantly up-regulated in the disease model group (ratio > 1.3, *p* < 0.01) but decreased in the RuXian-I group. 

**Figure 3:**
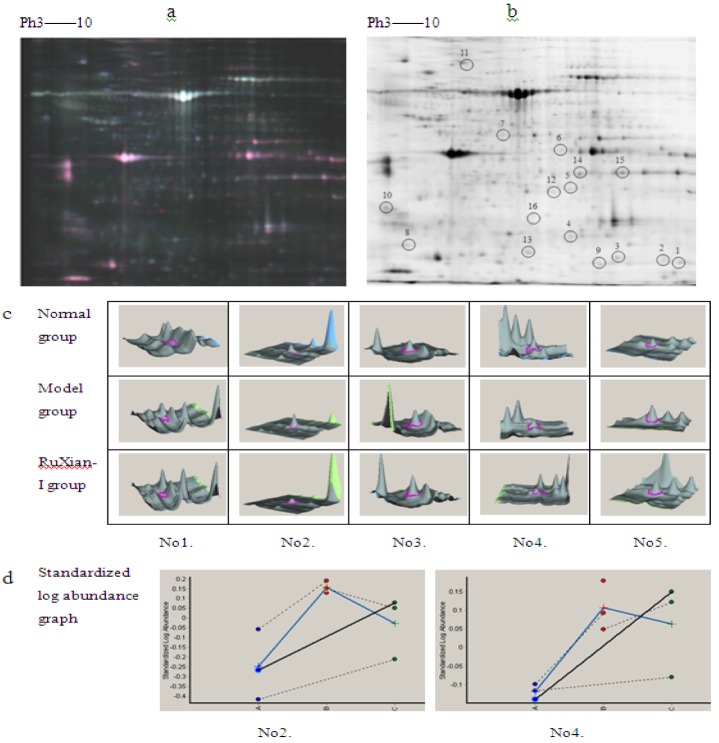
2D DIGE images and differentially expressed protein spots pictures of DeCyder software analysis. Figure 3a shows 2D DIGE images. Proteins from model group proteins labeled with Cy5 are shown in red, control group proteins labeled with Cy3 are shown in green, and the blue spots are internal standard proteins labeled with Cy2. Figure 3.b. is an analytical gel image. The labels indicate 16 gene products that are altered upon RuXian-I treatment. The label represents the protein spots displayed in Table 1. Figure 3.c. shows 3D pictures for five proteins across the different groups. Panels c1 to c5 represent alpha B-crystallin, annexin A1, glutathione S-transferase P, superoxide dismutase [Mn], and Peroxiredoxin -1, respectively. Figure 3.d. shows 2 proteins’ Standardized log abundance graph. The solid blue line represents the average protein abundance change in the different groups (A, control group; B, model group; C, RuXian-I group). The *t-* test was used for statistical analysis of the data using Decyder biological variation analysis software.

On the other hand, there were five spots that were down-regulated in the disease model (ratio > 1.3, *p* < 0.01) but up-regulated after treatment with RuXian-I. One of the 2D DIGE images is shown in Figure 3a, and one of 2D DIGE analytical gels images are shown in Figure 3b. 3D pictures from DeCyder software analysis (for five representative spots) are shown in Figure 3c (No. 1–5) and standardized log abundance graph(for two representative spots) are shown in Figure 3d.

#### 2.3.2. Identfication of Differentially Expressed Proteins

Out of 27 spots, 17 corresponding to 16 different gene products were identified by matrix assisted laser desorption/ionization time-of-flight mass spectrometry (MALDI-TOF/TOF-MS). Protein spots are shown in Figure 3b. There are two spots (corresponding to Tpil protein and myosin-4) that are significantly down-regulated in the disease model (ratio < 1.3, p < 0.01) but increased after treatment with RuXian-I. The other 15 spots were significantly up-regulated (ratio > 1.3, p < 0.01) in the disease model group, but down-regulated after treatment with RuXian-I. The information on the 17 spots is listed in Table 1. Following protein identification, they were categorized using the PANTHER gene ontology database (http:// pantherdb.org). The PANTHER classification system grouped the 17 proteins from the mammary glands into 10 major subcategories according to “biological processes” is shown in Table 2, including the immune system process, metabolic process, response to stimulus, transport, *etc.*

We analyzed the mammary gland proteomes of the control rats, the disease model rats, and the RuXian-I-treated rats (n = 3 randomly selected rats per group) using the 2D DIGE technique. Among the differentially expressed protein spots, 16 gene products were identified by MALDI-TOF-MS. The following protein spots, which were altered upon RuXian-I treatment, are associated with the occurrence of breast cancer and metastasis.

**Table 1:** Identification results of differentially expressed proteins from breast tissues as identified by MALDI-TOF/TOF MS analysis.

NO.^a^	Protein description	Protein ID	Protein level^b^	Nominal **	Percentage	Mascot Score^e^	Molecular function
				Mr(kDa)/p *I^c^*	coverage (%)^d^		
1	Peroxiredoxin-1	Q63716	↑	22.07/8.34	35	197	oxidoreductase activity and peroxidase activity
2	Superoxide dismutase [Mn]	P07895	↑	24.659/8.96	41	108	oxidoreductase activity
3	Tpi1 protein	P48500	↓	26.701/7.07	43	112	isomerase activity
4	Glutathione S-transferase P	P04906	↑	23.424/6.89	39	117	transferase activity
5	Omega-amidase NIT2	P30919	↑	30.682/6.9	35	156	hydrolase activity
6	Glutamine synthetase	P09606	↑	42.24/6.64	25	103	ligase activity
7	Xaa-Pro dipeptidase	Q510D7	↑	54.71/5.61	42	136	peptidase activity, DNA binding, transcription factor activity
8	Translationally-controlled tumor protein	P63029	↑	14.949/5.09	41	102	structural constituent of cytoskeleton and cytoskeletal protein binding
9	Alpha B-crystallin	P02511	↑	19.945/6.84	66	316	structural molecule activity
10	Tropomyosin alpha-4 chain	P09495	↑	28.492/4.66	47	107	motor activity and structural constituent of cytoskeleton
11	Myosin-4	Q29RW1	↓	222.7/5.58	16	118	motor activity, structural constituent of cytoskeleton, protein binding and small GTPase requlator activity
12	Complement component 4	Q6MG90	↑	192.00/6.62	9	156	receptor binding and peptidase inhibitor activity
13	Immunoglobulin kappa constant region	XP575527	↑	23.42/5.94	39	142	antigen binding
14	Annexin A1	P07150	↑	38.805/6.97	71	417	calcium ion binding, calcium-dependent phospholipid binding
15	Annexin A2	Q07936	↑	38.6/7.55	62	527	calcium ion binding, calcium-dependent phospholipid binding
16	Beta-casein	P02665	↑	25.353/6.04	19	88	

^a^ The numbers indicate the spot positions in 2D gel as shown in Figure 3b; ^b ^↑ up-regulation in model group rats, after treatment by the Mongolian down-regulation; ↓, down-regulation in model group rats and up regulation after treatment by the RuXian-I; ^c ^Calculated from the database entry without any processing; ^d^ Calculated by amino acid count; ^e ^Protein scores greater than 83 are significant(p < 0.05).

**Table 2:** Classification of differentially regulated proteins according to biological functions. ^a^

Biological functions	Protein description
immune system process	complement component 4; immunoglobulin kappa V region; alpha B-crystallin; translationally controlled tumor protein; glutathione S-transferase P; superoxide dismutase [Mn]; peroxiredoxin-1
metabolic process	
*protein metabolic process*	alpha B-crystallin; complement component 4; xaa-Pro dipeptidase; omega-amidase NIT2
*lipid metabolic process*	annexin A1; annexin A2
*nitrogen compound metabolic process*	glutamine synthetase
*cellular amino acid and derivative metabolic process*	glutamine synthetase
*carbohydrate metabolic process*	Tpi1 protein
*oxygen and reactive oxygen species metabolic process*	superoxide dismutase [Mn]; peroxiredoxin-1
response to stimulus	alpha B-crystallin; glutathione S-transferase P; complement component 4; immunoglobulin kappa V region
transport	annexin A1; annexin A2; Beta-casein; myosin-4
signal transduction	complement component 4; myosin-4; annexin A1; annexin A2
developmental process	immunoglobulin kappa V region; tropomyosin alpha-4 chain; myosin-4; annexin A2
cell motion	tropomyosin alpha-4 chain; annexin A1; annexin A2
cellular component organization	tropomyosin alpha-4 chain; myosin-4
cell cycle	myosin-4
muscle contraction	alpha B-crystallin; tropomyosin alpha-4 chain; myosin-4

^a^ The subset of 16 gene products was classified to their gene ontology groupings using the PANTHER classification system (www.pantherdb.org/).

*Annexin.* Annexin A1 and annexin A2 belong to the annexin superfamily. Annexin A1 has been better studied than annexin A2. Annexin A1 is a strong inhibitor of glucocorticoid-induced eicosanoid synthesis and PLA_2_ activity. Annexin 1 has important functions in a variety of cellular effects, including inflammatory signaling, cell proliferation, cell death signaling, phagocytic clearance of apoptotic cells, and carcinogenesis. Annexin A1 expression is increased in hormonally regulated tumors, such as breast [11] and pituitary tumors [12] However, Shen *et al*. demonstrated a significant reduction in Annexin A1 expression in ductal carcinoma *in situ* and invasive ductal carcinoma, compared to normal or hyperplastic tissues, by tissue microarray analysis. (The samples studied include normal and hyperplastic tissues, *in situ* and invasive tumors, and lymph node metastases, for a total of 1,588 cases.) Moreover, in benign breast tissue, myoepithelial cells showed strong expression of Annexin A1 [13]. Annexin A1 has antiproliferative activity in macrophages because of constitutive activation of the MAPK/ERK pathway [14].

*Proteins associated with antioxidants and stress.* Among the differentially expressed protein spots, there were some proteins that are correlated with redox regulation, signal transduction, and metabolism. Research has shown that estrogens can alter the expression of several antioxidant enzymes [15]. In our screen, we identified three proteins associated with antioxidants and stress, glutathione S-transferase P, superoxide dismutase [Mn], and peroxiredoxin-1. 

SOD converts the superoxide radical into molecular oxygen and hydrogen peroxide. Work by Parmara suggests that excess oxidative stress arising from loss of SOD function can arrest mammary gland maturation and induce epithelial hyperplasia with early premalignant features [16]. Estrogen can play a role in breast cancer initiation and progression through a mechanism that is tightly linked to oxidative damage, but the specific pathway mediating this effect is not clear. In estrogen-induced breast cancer, the expression of SOD was increased. SOD activity levels in the mammary tissue of rats treated with E_2_ for 240 days displayed a 3.1-fold increase relative to age-matched controls [27]. This ability of estrogen to induce oxidative DNA damage may be related to an ER-mediated mechanism [18]. Estrogens can increase mitochondrial ROS production by repressing uncoupling proteins and semiquinones derived from tissue-specific conversion of estrogen to catechol estrogen metabolites [19,20]. In this study, the changes in SOD activity suggest that oxidative damage has already occurred and that estrogen injection has stimulated the antioxidant defense system in the hyperplastic tissue. Thus, RuXian-I may have the ability to reduce oxidative damage. 

Peroxiredoxin-1 is a member of the peroxiredoxin family, which is widely expressed in prokaryotes and eukaryotes. Apart from its role in scavenging reactive oxygen species, peroxiredoxin-1 also has many other roles in processes such as epithelial cell proliferation and signal transduction [21,22,23]. Peroxiredoxin-1 regulates and promotes cell proliferation through c-abl and c-myc, and is associated with reduced apoptosis [24,25]. 

Glutathione S-transferase P1 (GST P1) is a member of the cytosolic GST superfamily. It can catalyze the glutathione conjugation of a variety of electrophilic xenobiotics, including environmental toxins, drugs, and endogenous cellular electrophiles [26]. Inactivation of GST P1 may make cells more susceptible to mutations and damage as a result of exposure to electrophiles, oxidative stress, and estrogen metabolites [27]. GST P1 has been shown to function as a regulator of mitogen-activated protein kinases (MAPK) through its nonenzymatic, ligand-binding activity. Variations in the expression and activity of GST P1 occur in various human cancers [28,29]. However, a recent quantitative analysis revealed no biologically significant methylation in peritumoral or hyperplastic breast tissue [30]. In this study, GST P1 expression is increased in the disease model group. This may be because GST P1 expression is increase upon intracellular oxidative stress and up-regulation of estrogen metabolites. In this study, these three cell defense-related enzymes were increased in the disease model group compared with the normal group and decreased after treatment with RuXian-I. This implies that RuXian-I may exert its therapeutic effects on hyperplasia by reducing the production or increasing the removal of cytotoxic substances.

*Heat shock protein.* α B-crystallin (HspB5) is one of the members of the mammalian small heat shock protein family [31]. It has two main roles. First, α B-crystallin expression is induced upon exposure of cells to stressors such as heat shock and reactive oxygen species, whereupon it exerts cytoprotective effects by functioning as a molecular chaperone [32]. Second, α B-crystallin inhibits apoptosis in response to many different stimuli, including chemotherapeutics, TNF-α, and reactive oxygen species [33]. α B-crystallin also plays a the potential pathogenic role in basal-like breast cancer, because B-crystallin overexpression promotes the transformation of immortalized breast epithelial cells and renders them tumorigenic in orthotopic xenograft models [34]. An association was observed between high levels of α B-crystallin in primary breast carcinoma and lymph node metastasis [35]. In the case of a-crystallin, a study has shown that disorganization of microfilament or microtubule networks results in the activation of pathways converging upon the MAPK p38 [36].

Several of the differentially expressed proteins identified in this study, e.g., annexin A1, GST P1 and α B-crystallin, are related to MAPK pathways, and others are related to breast cancer occurrence and metastasis. Moreover, the elevations in annexin A1 expression and SOD activity were verified. This suggests that the mechanism of RuXian-I treatment may involve regulation of the MAPK pathway. These data also provide new clues about the relationship between breast hyperplasia and breast cancer. After treatment with RuXian-I, annexin A1 expression was significantly lower than that in the untreated rats with hyperplasia, suggesting that it may be a relevant target of RuXian-I treatment in breast hyperplasia. RuXian-I regulates proteins associated with breast cancer, suggesting that RuXian-I may have a therapeutic effect in this disease; however, this hypothesis requires further verification. Our previous understanding of the etiology of mammary gland hyperplasia was that it was mainly caused by an imbalance between estrogen and progesterone signaling in breast tissue. However, no changes in estrogen receptor expression were detected in this study. This may be because the study was limited by the number of samples and existing micro-protein detection constraints. Moreover, in this study, not all of the protein spots that differed between the control and disease model groups but were unaffected by RuXian-I treatment were analyzed. These spots may be related to the pathogenesis of mammary gland hyperplasia. The relationship between estrogen or its receptor and the proteins identified in this research requires further study.

### 2.4. Protein Validation

To verify the DIGE results, we chose the protein annexin A1 for authentication by immunohistochemistry. A SOD activity assay was carried out using a commercially available kit on rat breast homogenate.

**Figure 4:**
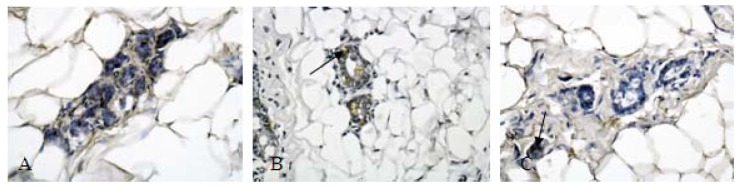
Immunohistochemical analysis of annexin A1 expression in rat besast tissue. (Magnification ×400; A, normal control group; B, model group; C, RuXian-I group). The arrows indicate the target proteins stained in brown-yellow. Statistically, positive expression rates of annexin A1 were significantly correlated with breast hyperplasia. After treatment RuXian-I, annexin A1 expression decreased. The arrow in C shows negative immunoreactivity of RuXian-I group.

**Figure 5:**
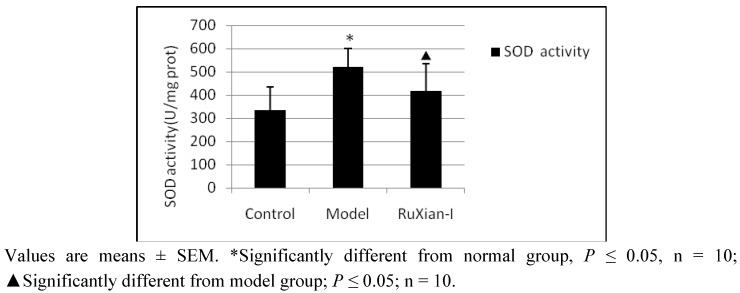
SOD activity assayed with commercial kits in rat breast tissue homogenate in various groups.

A representation of the anti-annexin A1 Immunohistochemical for the normal, breast hyperplasia, and treated by RuXian-I from one of each of these tissue cases is shown. As seen in Figure 4, the polyclonal anti-annexin A1 antibody showed strong anti-annexin A1 staining in the model group compared to normal group and reduced annexin A1 expression in RuXian-I group compared to model group. This pattern of annexin A1staining reactivity was identically observed in the other rats’ breast tissue of each group. The enzyme activity of SOD also was measured in rat breast tissue homogenate. Figure 5 shows a significant increase in SOD activity in rat breast hyperplasia model that be copied using estrogen and progesterone intramuscular method. But SOD activity decreased in RuXian-I group compared with model group.

## 3. Experimental

### 3.1. Reagents

Estradiol benzoate injection and progesterone injection were bought from Shanghai Tongyong Pharmaceutical Company Limited, Tamoxifeni citras was made in Sunray Pharmaceutical Co., Ltd (Suzhou, China).All reagents were of analytical grade and were purchased from Amersham Biosciences (Uppsala, Sweden). The ProteoExtract® Protein Precipitation Kit was from Merck (Darmstadt, Germany). The rabbit anti-annexin A1 polyclonal antibody was purchased from Boaosen Biotechnology Corporation (Beijing, China). The ready-to-use SP immunohistochemical study kit and the goat HRP secondary antibody against rabbit IgG were obtained from Beijing fir Zhongshan Goldenbridge Biotechnology Corporation. The kit for determining SOD was purchased from the Jiancheng Institute of Biotechnology (Nanjing, China). Female Wistar rats were from the Jilin University Laboratory Animal Center. 

### 3.2. Preparation of RuXian-I

RuXian-I was provided by the Mongolian Medicine Manufacturing Room of the Affiliated Hospital of Mongolia University for the Nationalities. The components of RuXian-I are listed in Table 3. Firstly, the crude herbs were crushed into small pieces. Then these small pieces were processed by one of following two methods: (1) extracting with distilled water and then filtering, concentrating and evaporating the extract, leaving a dry powder; (2) crushing by ball milling and sifting to obtain a fine powder. The two kinds of powder were properly mixed and pills prepared by rolling a soft mass into a round shape. After drying, coating, polishing, sterilization and packaging, the product was considered complete. The product (5 g) was powdered dissolved in distilled water (75 mL). The samples were shaken well before use.

**Table 3:** The components of RuXian-I.

Raw materials ^a^ and dose			
Latin name		Medicine site	Dose
Raw materials processed by extracting with distilled water ^a^			
(1)	*Fructus Amomi Rotundus* (Zingiberaceae)	Fruit	250.0 g
(2)	*Herba Leonuri Japonici* (Labiatae)	Whole herb	75.0 g
(3)	*Flos Caryophylli* (Myrtaceae)	Alabastrum	75.0 g
(4)	*Fructus Gardeniae* (Rubiaceae)	Fruit	50.0 g
(5)	*Rhizoma Gymnadeniae Conopseae* (Orchidaceae)	Stem tuber	50.0 g
(6)	*Semen Myristicae* (Myristicaceae)	Kernal	50.0 g
(7)	*Lignum Aquilariae Resinatum* (Thymelaeaceae)	The wood contains Resina	50.0 g
(8)	*Fructus Hippophae* (Elaeagnaceae)	Fruit	50.0 g
(9)	*Thladiantha Dubia Bge* (Cucurbitaceae)	Stem tuber	50.0 g
(10)	*Sorbaria Sorbifolia* (Rosaceae)	Kernal	50.0 g
(11)	*Fructus chebulae* (Combretaceae)	Fruit	25.0 g
(12)	*Rhizoma Kaempferiae* (Zingiberaceae)	Rhizome	25.0 g
(13)	*Flos Carthami* (Compositae)	Flower	25.0 g
(14)	*Radix Aucklandiae* (Compositae)	Root	25.0 g
(15)	*Inula Helenium L.* (Compositae)	Root	25.0 g
(16)	*Sabina chinensis* (Cupressaceae)	Fruit	25.0 g
(17)	*Faeces Trogopteri* (Petauristidae)	Feces	25.0 g
(18)	*Semen Cyclogalanopsis Glaucae* (Fagaceae)	Kernal	25.0 g
(19)	*Herba Artemisiae Scopariae* (Compositae)	Seedlings	15.0 g
(20)	*Lacca* (Lacciferidae)	Glue secretion in the branches	10.0 g
(21)	*Folium Eriobotryae Japonicae* (Rosaceae)	Leaf	10.0 g
(22)	*Radix Arnebiae* (Boraginaceae)	Root	10.0 g
(23)	*Trollius Chinensis Bunge* (Ranunculaceae)	Flower	10.0 g
(24)	*Radix Sophorae Flavescentis* (Leguminosae)	Root	10.0 g
(25)	*Fructus Toosendan* (Meliaceae)	Fruit	10.0 g
(26)	*Radix Rubiae Cordifoliae* (Rubiaceae)	Root	10.0 g
Raw materials processed by direct crushing ^a^			
(27)	*Calculus Bovis* (Bovidae)	Gallstone	5.0 g
(28)	*Cornu Cervi Pantotrichum* (Cervidae)	Immature horns	3.0 g
(29)	*Cordyceps* (Clavicepitaceae)	Stroma	2.0 g
(30)	*Borax* (Borate)	Mineral	2.0 g

**^a^** Dosage represents dry weight.

### 3.3. Rat model of breast hyperplasia and medications

Virgin female Wistar rats weighing 180–220 g were allowed to acclimate in our facility for 2 weeks. The rats were housed at a temperature of 21 ± 1 °C and 50 ± 5% relative humidity with constant light/dark periods of 12 h each. They had free access to rodent diet and tap water. Ten rats were randomly removed from thirty adult Wistar female rats as normal control group, and the remaining as disease model group. Normal rats were injected with normal saline (0.25 mL/kg) for 30 days. Disease model group rats were injected with Estradiol Benzoate (0.5 mg/kg) for 25 days, followed by progesterone (4 mg/kg, i.m.) for 5 days to copy. The rats in the disease model group were then randomly divided into two groups (ten rats in each group), the model group and the RuXian-I treatment group. RuXian-I treatment group was maintained on RuXian-I (1.0 g/kg, oral gavage), for 30 days. The control group and the model group were given normal saline (10 mL/kg, oral gavage) for 30 days. During the experiment, the rats’ nipple diameters were measured weekly. The University Animal Care and Use Committee approved all animal procedures.

### 3.4. Histopathological observation of breast tissue

Right breast tissues from each rat were fixed in 10% formalin and paraffin-embedded; 5-µm paraffin sections were prepared for hematoxylin and eosin staining (H&E) and examined under a Zeiss light microscope. H&E-stained histologic sections were reviewed by the study pathologist (Cai L), who was blinded to the randomly assigned treatments. The following features were scored on a scale of 1 to 4 (normal, mild, moderate, florid): acinar ectasia, duct ectasia, epithelial hyperplasia, ductal hyperplasia, inflammatory reaction, fibroplasia, and changes in secretion.

### 3.5. Protein sample preparation

The left mammary gland tissues were stored at −80 °C in a freezer for proteome analysis. Three rats’ mammary gland tissues were randomly selected from each group and pestled in liquid nitrogen then weighed. Subsequently, the tissues were placed in 1.5 mL centrifuge tubes and fully mixed in lysis buffer containing 30 mM Tris, 7M urea, 2M thiourea, 4% CHAPS, 1% nuclease and 1% protease inhibitor (volume ratio), and denatured 1 h at room temperature. Then protein samples were centrifuged for 30 min at 25,000 g at 15 °C. The supernatant was precipitated with precipitant (Merck) and re-dissolved with lysis buffer. Protein concentrations were determined using the Bradford assay, then all samples were stored at −80 °C.

### 3.6. Fluorescence labeling with CyDyes

Protein was labelled with the CyDyes Fluors for 2D-DIGE technology (GE Healthcare/Amersham Biosciences) according to the manufacturer’s recommended protocol. The pH of the protein samples was adjusted to 8.5 with NaOH (50 mM). Three group samples were randomly labeled with Cy3 or Cy5. Whereas internal standards a mixture of equal amounts of protein extracts from normal control group, model group and treatment group labeled Cy2. In each sample 50 µg of protein were labeled with using 400 pmol of fluorochrome. Labeling reaction was performed in the dark on ice for 30 minutes. End the reaction was by adding 1 µL of lysine (10 mM) in the dark on ice for 10 minutes. To each labeled samples 3 mg DTT, 1% IPG buffer was added, followed by rehydration buffer (7 M urea, 2 M thiourea, 2% CHAPS, a trace of bromophenol blue) supplement ed to 450 µL in the ultimate sample.

### 3.7. 2D-DIGE

450 µL hydration buffer containing the labeled sample were loaded onto a 24-cm Immobiline DryStrip gel (pH 3–10 nonlinear). Rehydration process includes 6 hours without voltage and 6 hours with 30 V voltage. Hydration and IEF were carried out on an Ettan IPG Phor system (GE Healthcare). Isoelectric focusing process is 200 V for 1 h, 500 V for 1 h, 1,000 V for 1 h, 8,000 V for 1h, and 8,000 V for 7 h in 20 °C. Following isoelectric focusing, the gel strips were equilibrated at room temperature, contains two steps, first, strips were equilibrated in equilibration buffer containing 50 mM Tris pH 8.8, 6 M urea, 30% glycerol, 2% SDS (F.w. 288.38), a trace of bromophenol blue, and 1% DTT for 15 min; the second, 1% DTT in equilibration buffer was replaced with 2.5% iodoacetamide for 15 min. The second-dimensional electrophoresis was carried out on a 12.5% (wt/vol) SDS-PAGE gel with 1-mm-thick, at 1.5W/gel on an Ettan DALT-12 separation unit (GE Healthcare). When the blue dye front reached the bottom of gel, the electrophoresis is ended.

### 3.8. Image acquisition and analysis

The gel was scanned using a Typhoon 9400 variable mode imager (GE Healthcare) to obtain images of labeled proteins. Different fluorescent dyes select corresponding wavelengths. Images were then analyzed using DeCyder Differential In Gel Analysis version 4.0 software (GE Healthcare) to identify spot fluorescence intensities. The estimated number of spots was set to 2,500. The DeCyder biological variation analysis module was used to detect protien spots and simultaneously match all 14 protein spot maps from five gels. All matches were also confirmed manually. Protein spots that were differentially expressed in control group and disease model group were marked (|ratio| ≥ 1.3, *p* ≤ 0.01). The *t-*test was used for statistical analysis of the data. Spots to be analyzed were picked from a separate preparative gel on which 600 µg each of control group and disease model group proteins had been run and stained for total protein using Coomassie brilliant blue staining.

### 3.9. In-gel digestion of proteins and MALDI-TOF/TOF-MS protein identification

Protein spots on Coomassie Brilliant Blue R-350 stained gels were excised and washed three times with ultrapure water. The gel pieces were destained twice with 25 mM NH_4_HCO_3_ in 50% (v/v) acetonitrile (ACN), dried with a centrifugal concentrator (Thermo Savant SpeedVac Concentrator, USA). The dried protein pellets were re-hydrated and digested with sequencing grade trypsin (Roche, Switzerland) for 1 h at 4 °C, then solution containing 25 mM NH_4_HCO_3 _was filled up in digestion for 15 h at 37 °C. After the trypsin digestion, the minced gel pieces were separated from the digestion solution and washed with 5% trifluoroacetic acid (TFA) in 50% (V/V) ACN for 1 h at 40 °C, and then 2.5% TFA in 50% (v/v) ACN for 1 h at 30 °C for further extraction of peptides. The peptides were concentrated using a centrifugal concentrator. For MALDI-TOF -MS, 1 µL of the digest was mixed with 2 µL of the matrix solution (5 mg α-cyano-4-hydroxycinnamic acid in 80% v/v acetonitrile and 0.1% w/v TFA) and 1 µL of this mixture was deposited onto the MALDI target. For MALDI-TOF MS analysis was carried out using a MALDI-TOF tandem mass spectrometer (Ultraflex^TM^ III, Bruker Daltonics). For acquisition of mass spectra, 0.5 µL samples were spotted onto a MALDI plate, followed by 0.5 µL matrix solution. Mass data acquisitions were piloted by flexcontrol™ Software v 3.0 using batched-processing and automatic switching. The MS spectra were calibrated using PeptideCalibStandard II^TM^ (Bruker Daltonics) resulted in mass errors of less than 50 ppm. Peptide precursor ions corresponding to contaminants including keratin and the trypsin autolytic products were excluded in a mass tolerance of ± 0.2 Dalton.

### 3.10. Immunohistochemical detection of annexin A1 and SOD activity assay

The sections from representative paraffin-embedded tissue samples were de-paraffinized, rehydrated, and incubated in 3% (vol/vol) H_2_O_2_ for 15 min, and then in primary antibody (annexin A1, rabbit anti-rat polyclonal antibody, 1:350 dilution) at 37 °C for 3 h. The slides were then incubated with anti-rabbit secondary antibodies for 40 min and HRP-labeled streptavidin peroxidase for 20 min. Activity was visualized by incubating the slides for 10 min with DAB (Zhongshan Corporation). The slides were then counterstained with Harris hematoxylin. Image-Pro Plus 6 image analysis software was used for semi-quantitative analysis of staining. The SOD activity assay was carried out using a commercial kit and rat mammary gland homogenate. The assays for total SOD activity were based on the ability to inhibit the oxidation of oxyamine by the xanthine–xanthine oxidase system [37].

## 4. Conclusions

In the present work, a combination of progesterone and estrogen was successfully used to model breast hyperplasia in rats. 2D-DIGE and MALDI-TOF-MS were used as proteomic tools, and the protein expression profiles of the control, disease model, and RuXian-I-treated rats were compared. Our work is the first attempt to study the mechanism of action of this traditional Mongolian remedy by proteomics. The 17 we identified are involved in various biological processes, including immune system regulation, metabolism, transport, and more. Moreover, some of the differentially expressed proteins are known to be involved in breast cancer progression and metastasis. Ideally, our results will not only provide clues in the search for the mechanism of the therapeutic action of RuXian-I in mammary hyperplasia, but also aid our understanding of the molecular mechanisms linking breast hyperplasia to breast cancer, enabling early prevention. 
